# First responders’ experiences and training needs at the scene of a suspected suicide in Ireland: A mixed methods study

**DOI:** 10.1371/journal.pone.0360382

**Published:** 2026-10-13

**Authors:** John Goodwin, Saoirse Duggan, Katerina Drakos, Patrick Cotter, Ryan Biskupovic-Goulding, Sean Kelleher, Maria O’Malley, Nuala Walshe, Brendan Noonan, Rachel Flynn, Emma Gleeson, Andrea Kelly, Lisa Culloty, Nell Fingleton, Omar Fitzell, John Cudmore, Owen Hackett, Victor Shine, Bruce Pierce, Carmel Crowe, Ger O’Dea, Aine O’Donovan

**Affiliations:** 1 School of Nursing and Midwifery, University College Cork, Cork, Ireland; 2 Child and Adolescent Mental Health Center, University Hospital-Mental Health Services CPH, Copenhagen, Denmark; 3 Copenhagen Center for Social Data Science (SODAS), University of Copenhagen, Copenhagen, Denmark; 4 School of Clinical Therapies, University College Cork, Cork, Ireland; 5 An Garda Síochána, Dublin, Ireland; 6 Cork City Fire Brigade, Cork, Ireland; 7 Royal National Lifeboat Institution, Kinsale, Cork, Ireland; 8 The Irish Coast Guard, Cork, Ireland; 9 Association of Clinical Pastoral Education, Dublin, Ireland; 10 Pieta, Dublin, Ireland; 11 National Ambulance Service, Cork, Ireland; University of Toronto, CANADA

## Abstract

**Background:**

First responders are categorised as those who are the first to respond to emergency calls and attend the site of an emergency. They include, but are not limited to police, emergency medical technicians, firefighters, and water safety personnel. First responders play a critical role in suicide postvention, supporting those bereaved by suicide and preventing adverse outcomes. However, many responders feel uncertain about how to effectively support those bereaved by suicide and often lack formal postvention training. This study aimed to explore and understand the experiences and training needs of first responders when attending the scene of a suspected suicide.

**Methods:**

A mixed-method approach was adopted. A national quantitative survey with first responders was conducted. The impact of previous suicide-related training on suicide literacy, attitudes, and postvention knowledge was analysed using multivariate ANOVA, adjusting for sociodemographic factors. Qualitative data were collected through focus groups and analysed using reflexive thematic analysis.

**Results:**

A total of 1587 first responders completed surveys. Participants demonstrated positive and non-stigmatising attitudes towards suicide prevention and suicidal individuals, though lower scores were achieved for suicide literacy, postvention knowledge, organisation support, and preparedness. Previous suicide-related training was significantly associated with higher literacy, attitudes, and postvention knowledge, although effect-sizes were small. Qualitative findings from 10 focus groups revealed substantial gaps in training and confidence when responding to suspected suicides. Participants also described the considerable emotional toll of attending the scene and emphasised the need for support in the aftermath.

**Conclusion:**

Attending the scene of a suspected suicide has an impact on first responders. Limited suicide prevention and postvention training creates challenges, particularly in communicating with the bereaved. These findings underscore the need for comprehensive suicide prevention and postvention training and the allocation of adequate support.

## 1. Introduction

First responders are defined as trained professionals and volunteers who provide immediate assistance during emergencies and are often the first to arrive at the scene following an emergency call [1]. They include, but are not limited to police, emergency medical technicians, firefighters, and water safety personnel [2–4]. Due to their role, first responders experience increased exposure to traumatic events, including suicide [5]. Indeed, their exposure to suicide is significantly higher than the general population, with research showing police officers have on average 30 suicide exposures during their career [6]. This high exposure is not exclusive to police officers, with firefighters reporting on average 13.1 suicide exposure [7], and paramedics also cited to have a high frequency of exposure to suicide [8]. In the Irish context, there remains limited research of first responders’ exposure to suicide; however, in terms of national rates, in 2022 there was a standardised rate of 8.6 suicide deaths per 100,000 [9]. This figure highlights the potential for a high exposure to suicide among Irish first responders.

Repeated occupational exposure to suicide has been associated with adverse psychological outcomes among first responders, such as increased stress, sleep disturbances, substance misuse, post-traumatic stress disorder, and depression [1,10,11]. Rates of suicide/suicidal ideation have also reported to be higher among some first responder groups than in the general population, whereby firefighters have reported a suicide ideation rate as high as 48.8%, and emergency medical technicals have a tenfold increased risk of suicide compared to the general population [12]. Evidence suggests that responding to the scene of a suspected suicide can be emotionally and psychologically challenging for first responders [13,14]. Despite this, the experiences of first responders are often overlooked in the workplace, with opportunities to debrief being rare [15].

While first responders routinely encounter a wide range of traumatic incidents, responding to the scene of a suspected suicide presents additional emotional challenges. First responders are often among the first professionals to interact with bereaved family members [16]; such encounters require balancing procedural duties with compassionate communication, often in uncertain circumstances, with many first responders reporting feeling unprepared [13,16]. As such, attending suicide scenes present unique challenges for first responders.

First responders typically interact with the bereaved in the first 72 hours after a death [16] and play a critical role in suicide postvention. Postvention is defined as “those activities developed by, with, or for suicide survivors, to facilitate recovery after suicide, and to prevent adverse outcomes including suicide behaviour” [17]. Including timely and appropriate postvention measures in the aftermath of a suicide can reduce morbidity and mortality and assist with the healing process of families and communities [16,18]. Recently, Ireland’s 2026–2036 strategy to reduce suicide and self-harm, *Connecting for Life*, was launched, including reference to first responders’ involvement in the development of clear notification pathways following a suicide, and their role in support and signposting for the bereaved. This highlights the importance of first responders’ in Ireland role in postvention [19]. Despite the impact which postvention measures can have, first responders often feel uncertain on how to effectively support those bereaved by suicide and often lack formal postvention training [13,16], highlighting the need for targeted postvention training.

Sufficient training for first responders has the potential to increase the efficiency of suicide responses [20], with training associated with improved understanding of suicide and reductions in stigma. However, many first responders’ express dissatisfaction with their current suicide training which is considered to be minimal, unspecific, and deprioritised [21].Additionally, suicide training for first responders varies across the international context, with no international standards for training [22]. While little research explores these differences across first responder professions, a recent scoping review of suicide prevention training for allied health professionals found the evidence is concentrated across a small number of countries [23]. This therefore limits the understanding of how training and outcomes translate across different professions and cultures. Considering the importance that suicide and suicide postvention training has on first responders’ ability to efficiently respond to the scene of a suicide, there is a need to develop a specific and prioritised postvention training programme for first responders when attending the scene of a suspected suicide. Therefore, the aim of this study is to explore what are the training needs of first responders when attending the scene of a suspected suicide and to understand what are the experiences of first responders when attending the scene of a suspected suicide, with particular attention on postvention training requirements.

## 2. Materials and methods

### 2.1. Study design

This study employed a convergent parallel mixed methods design, in which quantitative and qualitative data were collected using a staggered overlapping timeline, analysed separately, and then integrated during interpretation. Integration was achieved through comparison of quantitative and qualitative findings during the interpretation phase Findings from both strands were compared to identify areas of convergence and divergence [24]. Quantitative findings provided an overview of first responders’ suicide literacy, attitudes, preparedness, and postvention knowledge, while the qualitative findings were used to contextualise these findings. The integrated interpretation informed the overall conclusions of the study by considering how the two strands complemented one another [25].

### 2.2. Sample and setting

#### 2.2.1. Data collection.

Online surveys were conducted using Qualtrics [26] with data collection occurring in January 2025 through till June 2025. Surveys were disseminated through the networks of a National Steering Group, consisting of members of the Health Service Executive (HSE) National Office for Suicide Prevention (NOSP), and of first responder organisations (An Garda Síochána [Irish Police force] [AGS], National Ambulance Service [NAS], and Fire Service). The study was also advertised via social media. Participants were eligible to take part if they were a member a first responder group in Ireland.

At the end of the survey, interested participants were invited to email the researchers to partake in an online multi-disciplinary focus group. Information about the focus groups was also disseminated by the Steering Group and advertised on social media. These were conducted online vis MS Teams and facilitated by two to three researchers from June 2025 to September 2025. The duration of focus groups ranged from 68 to 95 minutes. The focus groups were designed to have mixed disciplines of first responders for the purpose of maximum variation [27]. No participant withdrew their data post-participation in the focus group.

#### 2.2.2. Ethical considerations.

Ethical approval for the study was obtained from the relevant bodies (Social Research Ethics Committee, University College Cork, Log: 2024−096; HSE Reference Research Ethics

Committee Midlands Area and Corporate Division [HSE Dublin & Midlands], Log: RRECB0225AOD; Garda Research Review Board). Informed written consent for both survey and focus groups was obtained using an online Qualtics form, prior to participation. All participants were given the right to withdraw their response up to two weeks post-survey completion or participation in a focus group. Confidentiality was discussed by the researchers prior to commencing the focus group and reiterated at the end. Due to the sensitivity of the topic, participants were provided with available supports after completion.

#### 2.2.3. Measurement tools.

Suicide literacy was assessed using the 26-item Literacy of Suicide Scale (LOSS) [28]. The item can be divided into four subscales: signs of suicide, risk factors, cause of suicide, and treatment. All 26 items gave response options of true, false, or don’t know, with total scores ranging from 0 to 26. The scale had an internal consistency of α = 0.71.

Suicide postvention knowledge was measured using the Knowledge of Suicide Postvention Scale (KSPS) [29]. It is a 21-item scale, with responses of true, false, and don’t know, with total scores ranging from 0 to 21 and an internal consistency of α = 76.

To assess attitudes towards suicide, the 12-item Occupational Suicide Attitudes Questionnaire (OSAQ) was used [30]. The scale measured the extent to which occupational staff hold positive and non-stigmatising attitudes towards suicide prevention and suicidal persons, alongside their confidence in their organisation’s ability to support them. The scale has three subscales: attitudes towards suicide prevention, attitudes towards suicidal persons, and organization self-efficacy. Questions are rated on a four-point Likert scale (“do not agree at all” to “agree completely”), with total scores ranging from 12 to 48 and with higher scores indicating more positive and non-stigmatising attitudes. The internal consistency of the scale was α = 0.69. As this value is marginally below the 0.7 threshold, the associations may be underestimated and findings should therefore be interpreted with caution.

Lastly, first responders’ preparedness to attend the scene of a suspected suicide was assessed using a seven-item author derived five-point Likert scale (“strongly disagree” to “strongly agree”), with scores ranging from 1 to 5 and had an internal consistency of α = 0.85. This scale has not undergone pilot testing or formal psychometric validation and should therefore be considered an exploratory measure.

#### 2.2.4. Data analysis.

Quantitative survey data was analysed using IBM SPSS 29.0 and RStudio 4.5 [31,32]. Participant characteristics were presented as proportions and scale scores were presented as mean and standard deviation (SD). One-way ANOVA and Multivariate ANOVA analyses were used to compare means of suicide literacy, attitude, preparedness/support, and postvention knowledge scales based on whether the participant had completed suicide related-training. Suicide-related training consisted of any training which had a focus on suicide including suicide prevention, mental health training, postvention training, etc. Results of the ANOVA are presented in tabular format as mean, SD, p-value (P), effect size (η²), estimated marginal means (EMM), and standard error (SE). Post-hoc pairwise comparisons were conducted using Games-Howell for unadjusted analyses and Bonferroni-corrected pairwise comparison of EMMs for adjusted analyses.

Focus group data were analysed using reflexive thematic analysis [33], with the process facilitated by NVivo [34]. Analysis was guided by an ontological relativist position, whereby the researchers acknowledged the existence of multiple realities and recognised that “truth” is contextually bound [33,35]. Consistent with this ontological position, an interpretivist epistemology was adopted, recognising that knowledge is socially constructed and that researchers play an active role in interpreting data and constructing meaning [35].

Transcripts were read numerous times to aid immersion and then coded by one researcher (JG). The identified codes were organised into themes based on similarities, with consensus of five researchers (SD; NW; EG; RF; LC). The results of the thematic analysis are presented in a narrative format.

#### 2.2.5. Reflexivity.

Consistent with reflexive thematic analysis, the researchers acknowledge that knowledge is co-constructed between researchers and participants [33]. The qualitative analysis was led by JG, a mental health nursing academic with experience in suicide research and qualitative research methods. The qualitative analysis team comprised researchers with backgrounds in public health, nursing, psychology, social sciences, and speech and language therapy. Throughout the analytic process, researchers engaged in reflexive discussions to consider how their professional backgrounds, experiences, and assumptions might shape data interpretation and theme development. These discussions were used to challenge interpretations, explore alternative explanations, and deepen understanding of participants’ accounts [33].

## 3. Results

### 3.1. Quantitative results

#### 3.1.1. Participant characteristics.

A total of 1587 first responders with representation from all counties in the Republic of Ireland were included in this study; full breakdown is available in S1 File. Participants were predominately males (65.8%) aged between 30–59 (93.9%). Members of AGS accounted for over half of the sample (59.3%), followed by Community First Responders (CFRs) (15.7%), NAS (14.8%), and The Fire Service (9.6%). The remaining participants were primarily voluntary first responder groups including Voluntary Emergency Services (5.9%), Coast Guard (2.6%), Royal National Lifeboat Institution (RNLI) (1.4%) and Other (3.3%). The predominant response being from members of AGS reflects the service being the largest first responder group in Ireland, with approximately 14,000 active members [36], which equates to a 6.7% total response rate from AGS. Nearly 15% of responses (n = 235) were from NAS [37], which is responses from 10% of NAS members. The Fire Service accounted for 9.6% (n = 152) of responses [38], which translates to 5% of the active workforce.

In terms of employment, 79% were full-time workers with 59% having over 15 years’ experience in their role. Exposure to suicide was high among the participants with 93% reporting they had attended the scene of a suspected suicide and 47.3% had been affected by suicide of a family member or close friend. Nearly two thirds reported they had previously completed suicide-related training, with 35.3% completed training as a part of their mandatory professional training, whereas 28.2% had completed training as self-directed professional development. Table 1 describes the full survey participant characteristics.

**Table 1:** Survey participant characteristics.

Characteristics	Total = 1587 N (%)
**Age**	
*18-29*	89 (5.6)
*30-39*	336 (21.2)
*40-49*	719 (45.3)
*50-59*	387 (24.4)
*60+*	56 (3.5)
**Gender**	
*Males*	1044 (65.8)
*Females*	540 (34.2)
*Prefer not to say*	3 (0.2)
Profession*	
*An Garda Síochána*	941 (59.3)
*National Ambulance Service*	235 (14.8)
*The Fire Service*	152 (9.6)
*Royal National Lifeboat Institution*	22 (1.4)
*Coast Guard*	41 (2.6)
*Community First Responder*	249 (15.7)
*Voluntary Emergency Services*	94 (5.9)
*Other*	53 (3.3)
**Years of experience in role**	
*0-5*	245 (15.4)
*6-10*	273 (17.2)
*11-15*	109 (6.9)
*16-20*	377 (23.8)
*21+*	559 (35.2)
*Missing*	24 (1.5)
Employment status*	
*Full-Time*	1255 (79.1)
*Volunteer*	320 (20.2)
*Retained*	48 (3.0)
*Part-Time*	14 (0.9)
**Been affected by suicide (Family/friend)**	
*Yes*	750 (47.3)
*No*	804 (50.6)
*Prefer not to say*	33 (2.1)
**Attended the scene of a suspected suicide in their role**	
*Yes*	1476 (93.0)
*No*	111 (7.0)
**Previous suicide Training**	
*Mandatory suicide training*	560 (35.3)
*Non-mandatory suicide training*	448 (28.2)
*No training*	579 (36.5)

*Respondents could select more than one profession and therefore the ability to choose more than one employment status; consequently, individuals may appear in more than one category.

#### 3.1.2. Suicide literacy, attitudes, preparedness, and postvention knowledge.

Table 2 displays the mean scores for the scales and subscales used to measure first responders suicide literacy, attitudes, preparedness, and postvention knowledge. Participants had, on average, achieved moderate scores on the LOSS Total with a mean of 16.71 (±4.59) out of a maximum of 26. Among the literacy subscales, participants had the highest mean score for treatment of suicide (Mean (M)=3.42 ± 0.87 out of 4) and the lowest mean for signs of suicide (M = 2.53 ± 1.36 out of 5). Participants also achieved moderate OSAQ total scores (M = 32.74 ± 4.12 out of 48); however, high scores were achieved for positive attitudes towards suicide prevention and non-stigmatising attitudes towards suicidal persons [prevention (M = 12.54 ± 1.99 out of 14), persons (M = 12.62 ± 2.39 out of 14)]. The lowest score for attitudes was for the subscale of organisational self-efficacy where the mean score was 7.58 (±1.98) out of 14.

**Table 2:** Participant mean literacy, attitudes, preparedness, and postvention knowledge scale scores.

Scales	Mean ±SD
**LOSS (Literacy)**	
*Total*	16.71 ± 4.59
*Sign*	2.53 ± 1.36
*Risk Factors*	4.16 ± 1.55
*Causes*	6.60 ± 2.28
*Treatment*	3.42 ± 0.87
**KSPS (Postvention)**	
*Total*	11.81 ± 3.77
**OSAQ (Attitudes)**	
*Total*	32.74 ± 4.12
*Positive attitudes towards suicide prevention*	12.54 ± 1.99
*Non-stigmatising attitudes towards suicidal persons*	12.62 ± 2.39
*Organisational self-efficacy*	7.58 ± 1.98
**Preparedness**	
*Total*	2.91 ± 0.83

Participants had KSPS scores with an average score of 11.81 (±3.77) out of a maximum 21. Participants also disagreed they were adequately prepared when attending the scene of a suspected suicide, with an average score of 2.91 (±0.83) out of 5.

### 3.2. Impact of previous suicide-related training

Participants’ suicide literacy, postvention knowledge, attitudes towards suicide, and suicide preparedness was compared based on training type (mandatory training, non-mandatory training, and no training). Unadjusted associations are displayed in Table 3, and adjusted in Table 4.

**Table 3:** The effect of suicide training on suicide literacy, attitudes, preparedness, and postvention knowledge (unadjusted).

Scale	No Training M ± SD	Mandatory M ± SD	Non-Mandatory M ± SD	p	η²	p NT vs M	p NT vs NM	p M vs NM
**Loss (Literacy)**								
*Total*	15.70 ± 4.84	17.60 ± 4.14	17.00 ± 4.52	**<.001**	.033	**<.001**	**<.001**	.066
*Sign*	2.31 ± 1.38	2.72 ± 1.33	2.57 ± 1.35	**<.001**	.016	**<.001**	**.007**	.198
*Risk Factor*	3.85 ± 1.61	4.38 ± 1.45	4.26 ± 1.54	**<.001**	.023	**<.001**	**<.001**	.434
*Cause*	6.18 ± 2.36	6.94 ± 2.13	6.71 ± 2.26	**<.001**	.021	**<.001**	**<.001**	.226
*Treatment*	3.31 ± 0.94	3.54 ± 0.78	3.42 ± 0.88	**<.001**	.013	**<.001**	.123	.051
**KSPS (Postvention)**								
*Total*	11.60 ± 4.02	11.90 ± 3.50	12.00 ± 3.73	.165	.002	.279	.219	.959
**OSAQ (Attitudes)**								
*Total*	32.10 ± 3.85	33.00 ± 4.33	33.20 ± 4.08	**<.001**	.010	**<.001**	.905	**.006**
*Attitude towards suicide prevention*	12.40 ± 1.88	12.60 ± 2.10	12.60 ± 1.98	.120	.003	.614	.070	.420
*Attitude towards suicide persons*	12.60 ± 2.37	12.50 ± 2.49	12.80 ± 2.26	.065	.003	.710	.234	.051
*Self-efficacy*	7.14 ± 1.83	7.95 ± 2.04	7.68 ± 1.99	**<.001**	.031	**<.001**	**<.001**	.094
**Suicide Preparedness Scale**								
*Total*	2.66 ± 0.75	3.19 ± 0.80	2.89 ± 0.85	**<.001**	.073	**<.001**	**<.001**	**<.001**

M = Mean; SD = Standard Deviation; η² = eta squared. p = overall ANOVA p-value. NT = No Training; M = Mandatory; NM = Non-Mandatory. Post-hoc comparison using Games-Howell. Cases included in analysis n = 1,587.

**Table 4:** The effect of suicide training on suicide literacy, attitudes, preparedness, and postvention knowledge (Adjusted for age, gender, years of experience in role, past suicide exposure personal and professional, and volunteer employment status).

Scale	No Training EMM ± SE	Mandatory EMM ± SE	Non-Mandatory EMM ± SE	p	η²	p NT vs M	p NT vs NM	P M vs NM
**Loss (Literacy)**								
*Total*	15.70 ± 0.27	17.50 ± 0.31	16.90 ± 0.31	**<.001**	.033	**<.001**	**<.001**	.106
*Sign*	2.43 ± 0.08	2.84 ± 0.09	2.71 ± 0.09	**<.001**	.016	**<.001**	**.005**	.364
*Risk Factor*	3.82 ± 0.09	4.25 ± 0.11	4.13 ± 0.11	**<.001**	.021	**<.001**	**.006**	.775
*Loss Cause*	6.14 ± 0.13	6.90 ± 0.15	6.67 ± 0.16	**<.001**	.021	**<.001**	**<.001**	.349
*Loss Treatment*	3.27 ± 0.05	3.51 ± 0.06	3.38 ± 0.06	**<.001**	.014	**<.001**	.155	**.047**
**KSPS (Postvention)**								
*Total*	12.10 ± 0.22	13.00 ± 0.25	12.80 ± 0.26	.161	.002	**<.001**	**.006**	1.000
**OSAQ (Attitudes)**								
*Total*	32.80 ± 0.25	34.00 ± 0.28	34.00 ± 0.29	**<.001**	.010	.120	1.000	**.031**
*Attitude towards suicide prevention*	12.60 ± 0.12	12.90 ± 0.14	12.90 ± 0.14	.071	.004	.096	**.006**	.881
*Attitude towards suicide persons*	13.00 ± 0.14	13.10 ± 0.16	13.40 ± 0.17	.064	.003	1.000	.054	.223
*Self-efficacy*	7.18 ± 0.12	7.97 ± 0.13	7.72 ± 0.14	**<.001**	.030	**<.001**	**<.001**	.135
**Suicide Preparedness Scale**								
*Total*	2.70 ± 0.05	3.30 ± 0.05	2.97 ± 0.06	**<.001**	.072	**<.001**	**<.001**	**<.001**

EMM = Estimated Marginal Mean; SE = Standard Error; η² = eta squared. p = overall ANCOVA p-value. NT = No Training; M = Mandatory; NM = Non-Mandatory. Post-hoc comparisons using Bonferroni correction. Cases included in analysis n = 1,530.

#### 3.2.1. Suicide literacy.

Training was significantly associated with an increase in suicide literacy both for total literacy and all subscales in both unadjusted (p=<0.001) and adjusted (p=<0.001) analyses, with effect sizes remaining low (η² = 0.01–0.03). For total literacy scores, mean scores rose form 15.70 (±4.84) for those with no training to 17.60 (±4.14) with mandatory training and 17.00 (±4.52) with non-mandatory, with the pattern remaining after adjustment (EMM = 15.70 ± 0.27, 17.50 ± 0.31, and 16.90 ± 0.31).

Post-hoc comparisons showed a consistent pattern, with for every subscale both mandatory and non-mandatory training groups scores significantly higher than no training groups (p=<0.001 to p = 0.007), with one exception. On the LOSS treatment subscale, non-mandatory training did not differ significantly for training in both unadjusted (p = 0.123) and adjusted (p = 0.155) models.

#### 3.2.2. Suicide postvention knowledge.

Training was not significantly associated with postvention knowledge in either the unadjusted (p = 0.165) or adjusted (p = 0.161) analyses, with a negligible effect size (η² = 0.002). Mean scores were similar across groups: 11.60 (±4.02) for no training, 11.90 (±3.50) for mandatory training, and 12.00 (±3.73) for non-mandatory training.

Similarly, unadjusted post-hoc comparisons revealed no significant pairwise differences between any of the three groups (p= > 0.21). However, the adjusted models pairwise comparisons indicated that both the mandatory (p= < 0.001) and non-mandatory (p = 0.006) training groups had significantly higher postvention knowledge than the no-training group after accounting for covariates.

#### 3.2.3. Attitudes towards suicide.

Training was significantly associated with increased total OSAQ scores. Unadjusted means were 32.1 (±3.85) for no training, 33.0 (±4.33) for mandatory training, and 33.2 (±4.08) for non-mandatory training (adjusted EMMs: 32.8 ± 0.25, 34.0 ± 0.28, and 34.0 ± 0.29). Post-hoc comparisons showed that both mandatory and non-mandatory training scored significantly higher than no training in the unadjusted analysis (p=< 0.001) and the adjusted analysis (p=< 0.0001 for both comparisons). Mandatory and non-mandatory training did not differ significantly from one another in either analysis (unadjusted p = 0.866; adjusted p = 1.000).

While training was associated with total OSAQ, it was not significantly associated with an increase in positive attitudes towards suicide prevention both in unadjusted (p = 0.12) and adjusted (p = 0.71) analyses. This finding was consistent with post-hoc test where there was no significant pairwise association apart from the adjusted- no training versus non-mandatory training being marginally significant (p = 0.45). Similarly, training was not significantly associated with non-stigmatising attitudes towards suicidal persons (unadjusted- p = 0.065, adjusted- p = 0.071), with no pairwise association in post hoc tests.

However, training was significantly associated with an increase of confidence in their organisations’ ability to support them in both the unadjusted (p= < 0.001, η² = .031) and adjusted (p=< 0.001, η² = .030) analyses. Unadjusted means increased from 7.14 (±1.83) with no training to 7.95 (±2.04) with mandatory training and 7.68 (±1.99) with non-mandatory training (adjusted EMMs: 7.18 ± 0.12, 7.97 ± 0.13, and 7.72 ± 0.14). Both mandatory and non-mandatory training scored significantly higher than no training in the unadjusted analysis (p=< 0.0001 and p = 0.0001) and the adjusted analysis (p=< 0.0001 and p = 0.0001). Mandatory and non-mandatory training did not differ significantly from one another in either analysis (unadjusted p = 0.094; adjusted p = 0.135).

#### 3.2.4. Suicide preparedness.

Training was significantly associated with increases in suicide preparedness both in the unadjusted (p = 0.001, η² = .07) and adjusted (p= < 0.001, η² = .072) analyses. Mean scores rose across the groups: 2.66 (±0.75) for no training, 3.19 (±0.80) for mandatory training, and 2.89 (±0.85) for non-mandatory training, with a comparable pattern in the adjusted EMMs (2.70 ± 0.05, 3.30 ± 0.05, and 2.97 ± 0.06). Additionally all pairwise analyses were statistically significant for all three groups.

### 3.3. Qualitative results

Ten focus groups were conducted, with each group comprising three to seven participants. Overall, 43 first responders participated in focus groups. Table 5 includes the breakdown of first responders’ roles. To protect participant confidentiality, only participants’ first responder role was recorded for the focus groups; additional demographic characteristics were not collected.

**Table 5:** Focus group participant first responder role.

Role	N (%)
*An Garda Síochána*	13 (30.2)
*National Ambulance Service*	9 (20.9)
*The Fire Service*	8 (18.6)
*Royal National Lifeboat Institution*	3 (7.0)
*Coast Guard*	1 (2.3)
*Community First Responders*	12 (27.9)
*Voluntary Emergency Service*	3 (7.0)

### 3.4. Themes

Five themes were identified: Responding to the Call: Preparation and Procedure; At the Scene: Challenges, Communication, Collaboration; The Emotional Impact of Suicide; The Spectrum of Support and Camaraderie; and Learning on the Job and Visions for Future Training.

#### 3.4.1. Theme 1: Responding to the call: Preparation and procedure.

This theme addresses how first responders prepare themselves to attend the scene of a suspected suicide, in addition to the practicalities associated with attending to the deceased. Participants spoke about receiving the call to attend the scene of a suspected suicide and the way they prepared themselves. Several participants commented on the importance of being physically fit, acknowledging the relationship between the body and the mind:

*“You have your own emotions to manage as well, which is effected by a whole load of different things from your state of fatigue, to hunger, to thirst, to how late you are into the shift*” (Focus Group [FG] 8, Participant [P]3, NAS)

Some volunteers and retained first responders had a different perspective on how they prepared. These participants spoke about the blurring of family life and their duty as first responders and how a call could mean having to disengage from their personal lives. In these instances, preparation was more about emotional detachment and disconnection:

*“I generally try not to have emotions and not to have feelings until I get to the situation because now I need to sit into my car I need to start the car, I need to get there safely”* (FG5, P1, CFR)

Others noted that “being prepared” is just not possible. Each scene attended presented its own nuances and no matter how comprehensive the information received was, unpredictability was inevitable, meaning it was not always possible to prepare for how one might respond physically or emotionally:

“*You’re never prepared, you can’t be prepared because you could go to the scene of two, maybe three [...], every case is different in the thirty years I’ve been on the Guards (AGS)”* (FG3, P5, AGS)

Participants emphasised the importance of maintaining a calm demeanour. They acknowledged the distress experienced by the deceased person’s family and friends and that keeping a “cool head” was vital to avoid amplifying intense emotions:

*“Don’t panic, don’t get caught up in the whole thing and, like, amplify the level of panic and whatever among the people you’re with”* (FG4, P1, CFR)

Several participants spoke about adhering to their clinical practice guidelines or policies and procedures that their organisation has in place. These provided structure for participants, simplified their duties, and ensured that they provided the most appropriate response at the scene:

*“You have to try to maintain your plan: ABCD and keep it as simple as possible”* (FG3, P4, NAS)

Moreover, having such clear structures in place allowed participants to enter “auto-pilot” mode and maintain composure. As such, performing their duties at the scene became quite task-based, with emotional detachment facilitating efficient decision-making:

*“It’s almost like an auto pilot: you’re not necessarily thinking about the person that you’re going to, you’re thinking about what has to be done”* (FG7, P2, AGS)

Participants emphasised the practical nature of their role at this point. The tasks here varied from using the knives they carried to cut down a rope, to retrieving the body of the deceased from a river:

*“Our role is more of a hands on practical one when it comes to suicide, whether be there to remove somebody from an object or for us which as regular occurrences in the river, whether it’s body retrieval or it’s a live rescue”* (FG6, P5, Fire Service)

This first theme provided an overview of how first responders prepared to attend the scene of a suspected suicide and how procedures guided them at the scene. Participants spoke about “preparedness” in the context of physical and mental domains, with many noting they had to emotionally detach. When at the scene, there was a sense that guidelines and procedures in place in each organisation were helpful, facilitating first responders to maintain a calm demeanour, with many referring to entering “auto-pilot” mode. These findings suggest that, while emotional preparedness was often perceived as unattainable, organisational procedures and task-focused practice provided first responders with a structured means of responding to the practical demands of suspected suicide scenes.

#### 3.4.2. Theme 2: At the scene: Challenges, communication, collaboration.

Under this theme, the way in which first responders interact with each other and with family members of the deceased is addressed. A common challenge participants faced was being the first to arrive on the scene. Waiting for other first responders to arrive was often a prolonged process, with reports varying from a half an hour to two hours’ waiting time:

*“Sometimes, you could be waiting a while; that’s the only thing that makes it quite difficult […], that you’re waiting so long, […] waiting an hour”* (FG9, P2, NAS)

Despite the challenges associated with waiting for colleagues to arrive at the scene, the consensus was that collaborations with other first responders were always very positive. There was a sense of common purpose among groups where individuals worked together to achieve the same goal:

*“There’s a common respect across all the rescue services […]: you’re all there for the same purpose”* (FG1, P1, RNLI)

Participants reported that role boundaries were very well defined across groups. This facilitated a safer scene, with the appropriate personnel attending to the relevant issue. Also, clear role definitions streamlined communication processes, as each first responder group could focus on their specific duty. Indeed, such was the cohesion between first responders and the appreciation and understanding of each other’s roles, that operating together at the scene often felt “choreographed”:

*“At the scene often there’s a bit of a choreography as to like, we’ll arrive first and because that’s our job and then the ambulance may arrive and then the fire service may arrive and then the Guards (AGS) may arrive […]. Everyone knows their bit of the dance”* (FG4, P1, CFR)

Whereas attending to the deceased was associated with policies and procedures which facilitated a sense of confidence in participants’ roles, interactions with families were reported as challenging due to a lack of guidance. Participants spoke about being bombarded by questions from distressed family members, and, while they wanted to respond with compassion, in many cases, participants expressed that they were uncertain how to answer:

*“There’s myriad questions that you don’t know how you’re going to answer them and you can’t tell them well, a lot of times you have to tell them that you don’t have the answer to that but they will just be coming to you, a perfect stranger in a lot of those situations and outpouring of grief and emotion”* (FG6, P2, AGS)

Participants who had completed training in the area of suicide prevention and postvention spoke very positively about their experiences. In particular, several participants commended the ASIST course and how, following completion, they felt more self-aware at the scene of a suicide in how they engaged with others:

*“I found that particular the Asist programme very, very important, and there’s times when I’m out on a call that I will remember […] I have to, you know, lower that tone and have a nice soft tone when I’m speaking, depending on the circumstances”* (FG5, P1, CFR)

Under the second theme, participants described strong collaborative relationships between first responder organisations, characterised by clearly defined roles, effective communication, and a shared sense of purpose. However, interactions with bereaved family members were consistently identified as the most challenging aspect of responding to suspected suicide scenes. Despite approaching these encounters with compassion and viewing them as a privilege, many participants felt underprepared and lacked confidence in their communication skills, fearing they might inadvertently increase distress. Those who had completed suicide-related training described greater confidence in engaging with bereaved family members.

#### 3.4.3. Theme 3: The emotional impact of suicide.

Under this theme, the impact of attending suicide scenes is presented. Participants spoke about their exposure to attending the scenes of suicides and the subsequent emotional impact. Most participants noted that they were exposed to such scenes very early on in their role as first responders:

*“I was only two weeks in the Guards (AGS) when I got my first suicide and I was, I suppose, how would you say, I was left to deal with a lot by myself”* (FG7, P4, AGS)

The volume of suicide-related calls was also discussed. Several participants had experiences of attending multiple suicide-related calls in the space of a single shift:

*“I’m plenty aware of people that would go to one incident at seven and could be at another similar incidence of death by half eight and it’s the next one it’s the next one it’s the next one, you know”* (FG3, P1, AGS)

Indeed, participants commented on how challenging it was to attend several suicide scenes in a row. Although participants were aware that they had important duties to fulfil and were obliged to attend calls when they were contacted by control, they were impacted by the inability to process what they had experienced at the scene:

*“One Sunday morning I attended three suicides one after the other”* (FG8, P1, NAS)

Participants acknowledged the responsibility they had in informing a family member that their loved one had died by suicide. Several participants recounted how bereaved family members expressed their anguish at the scene, and how this reaction haunted them long after:

*“I find that the most traumatic thing about the incident, not them actually taking their life but the reaction of the loved ones around: I find that is what I carry the most”* (FG4, P2, RNLI)

While there were different aspects of scenes that impacted on participants, there was consensus that attending the scene of a suicide takes its emotional toll on first responders. Because of adhering to strict procedures and entering “auto-pilot” mode at the scene, participants did not often receive an opportunity to process what they had experienced until after their shift was finished, meaning they often brought the burden home with them:

*“It’s when you drive home then you’d be thinking about thinking about their family and you know especially if you have own kids around that age and stuff”* (FG3, P3, CFR)

There was a sense that, after a time, the capacity to deal with suicide calls is eroded. Participants acknowledged that, while encountering suicides at the start of one’s career is certainly challenging, there is enough psychological resilience to cope. However, over time, and due to accumulated trauma, that resilience is gradually diminished:

*“I think every suicide, every fatality just takes a little bit of it, a chip out of your soul or a bit of a chip out of your spirituality or something”* (FG1, P4, Fire Service)

Consequently, participants suggested that first responders sometimes resign from their positions. First responders are not always prepared for the volume of suicide-related calls they are expected to answer to and are ill-equipped to process the associated trauma:

*“We do lose a lot of responders over suicides”* (FG5, P3, CFR)

This theme explored the emotional impact of attending suspected suicide scenes on first responders. Owing to the high volume of suicide-related calls, participants often felt there was little opportunity to process one incident before responding to the next, with many describing how the emotional burden extended into their personal lives. Participants also reflected on the cumulative impact of repeated exposure to suicide, describing a gradual erosion of resilience over time.

#### 3.4.4. Theme 4: the spectrum of support and camaraderie.

This theme addresses the supports, both formal and informal, that participants had either availed of, or believed should be available to them. There was a great deal of variance in terms of the supports participants were aware of. Peer support systems, for example, were considered to be inconsistent and, while efforts have been made to integrate peer support into various organisations, these had failed to sustain momentum:

*“There was talk in the past as well about peer supports there were years ago people kind fell out over it, it never took off again”* (FG10, P1, Fire Service)

Some participants noted that support was only ever offered in the event of a significantly traumatic event, or following multiple events. As such, some distressing call outs may not trigger intervention, indicating a lack of consistency around supports:

*“Yeah, so, it’s either severity or an accumulation, so, like, for us, probably every 3rd or 4th fatality, we bring those guys [counsellors] in and we have that one hour chat and a cup of tea”* (FG1, P4, Fire Service)

Supports were also discussed in the context of post-incident reflective practices, including diffusions, and debriefs, with different organisations adopting different approaches. No matter which form these post-incident reflections took, their importance was regarded to be paramount in reducing the burden of the trauma brought home:

*“I feel over the years debriefing, it’s huge for anything, even the smallest type of incident, without a debrief it can lead to people bringing it home with them and thinking about it and overthinking and, ah, little questions that could be ironed out”* (FG2, P3, Coast Guard)

Participants spoke about openness to seeking support. Although there was a sense that societal conversations about mental health had evolved, meaning there was less stigma about first responders reaching out for help, certain stigma related barriers were still evident. Several participants referred to traditionally male organisations and perpetuating male stereotypes which often impeded support seeking:

*“I know lads here would be kind of too hard to talk about it”* (FG6, P5, Fire Service)

Indeed, several participants conveyed their awareness of their organisation’s mental health strategy and the supports that were available should they be needed. However, they advised that, while supports have been accessible to first responders, encouraging them to access supports is negatively influenced by personal and social barriers:

*“There is options, but you know, it’s up to people to access them as well”* (FG4, P4, AGS)

Moreover, the way in which supports were offered to participants was considered tokenistic, with many using the phrase “tick the box” when speaking about supports. When participants felt they needed support and required human-to-human interaction, they were instead provided with a list of supports to contact, which was viewed as inauthentic:

*“It’s just that management can when the incident is up on the screen, were the members offered peer support? Tick. Were they offered welfare? Tick. Were they offered Inspire? Tick”* (FG3, P5, AGS)

Where formal supports were perceived as insufficient, or where there was a reluctance to attend available supports, participants described the importance of informal peer-to-peer supports. These often occurred organically, with conversations being sparked through shared experiences of attending traumatic scenes. There was also a sense of reciprocity, where participants supported their colleagues at one point, but later found themselves in need of similar support. This informal mutual support system fostered solidarity among participants, acting as a coping mechanism that was often perceived as more accessible than formal support:

*“Our unit, your group that whoever you’ve been working with that day, they’re your support first of all and you’d always have a chat afterwards”* (FG4, P3, AGS)

There was a sense of abandonment communicated, where the role of first responders in suicides scenes was not fully acknowledged; this left participants feeling invalidated and emotionally isolated. It was clear that basic acknowledgement and empathetic communication from management/supervisors was required to validate participants and reinforce their sense of belonging within the organisation:

*“We cut this young man down, we waited on the Guards (AGS) and we did everything that has to be done cause it’s a scene of crime and everything else and everything was done properly and then nobody sort of said to us “Are you OK guys?” you know, “Do you need a bit of downtime?*” (FG8, P1, NAS)

One participant emphasised the importance of systemic support, which would involve a shift in organisational culture, where help-seeking becomes the “norm”. It was suggested that embedding supports within an organisation in this fashion could transform supports from ad-hoc measures to proactive, structured approaches:

*“I think the support starts well before any incident happens and I think it depends on the culture in the organisation. […] If you build a culture where support is accepted and something that you know you should access if you need it and recognise when you need it: I think that needs to be first there”* (FG4, P2, RNLI)

This theme addressed the formal and informal supports available to first responders following attendance at suspected suicide scenes. Participants described considerable variation in both the availability of support and perceptions of its usefulness. While some valued the supports provided by their organisations, others viewed them as tokenistic or “tick-the-box” exercises. Participants also acknowledged that, despite support services being available, cultural and personal barriers could discourage help-seeking. Consequently, many advocated for a broader organisational culture in which compassionate, proactive support is routinely embedded into practice. Across organisations, post-incident reflections, including debriefs and diffusions, were consistently viewed as valuable opportunities to process experiences and support colleagues.

#### 3.4.5. Theme 5: Learning on the job and visions for future training.

The final theme addresses the training suggestions from first responders in relation to suicide postvention. Participants often felt out of their depth when supporting families at the scene of a suicide, with many noting they had never received any training in this area. However, participants spoke about becoming more comfortable engaging with families over time, drawing on their wealth of practical experiences:

*“I’ve just learned to sit with them in that space where they need time, they need to take it all in and there will be tears and there will be hurt and pain”* (FG8, P1, NAS)

This process of “learning as you go” was echoed by several participants. Learnings were taken from experiences at a scene and, where appropriate, participants recalled having to make adaptations to their approach, depending on the situation, often without certainty that the right course of action was being taken:

*“I feel confident, but I’m twenty-seven years after it. Now did I feel confident at the start? No. […] It’s like flying a plane and learning how to build it at the same time: you only gain it by doing it”* (FG9, P4, AGS)

Learning from other first responders was greatly valued by participants. They described how they would observe their more experienced peers engage with family members, noting the communication skills employed to convey empathy. These observations were internalised and shaped how participants themselves interacted with family members:

*“I took a lot of my experience from my peers and how they do it”* (FG9, P2, NAS)

As such, participants from all organisations suggested that training around communication skills is paramount. Participants felt that, by equipping them with enhanced communication skills, they would be more confident engaging with bereaved family members, thus providing more empathetic and effective support:

*“I think there should be more training on how to deal with the family members after somebody has taken their own life by suicide: that could be a big thing”* (FG3, P4, NAS)

Several participants spoke about their fear of saying “the wrong thing” to family members and how they could potentially amplify distress. Consequently, participants expressed a need for practical guidance, or a resource informed by previous encounters on “what to say” and “what not to say”:

*“I like the idea of […] what are good things to say just to somebody who wouldn’t have maybe the wherewithal within themselves. […] If you had phrases – useful words, what to say”* (FG2, P2, AGS)

Breaking bad news training was highlighted as an area of importance. Participants reflected on times when they broke bad news to a bereaved family member and wondered if they could have done so more tactfully:

*“I’ve wondered, “Did I do this correctly? Did I make the situation worse?”, so I went in and researching myself but definitely would be an area that we would love to see some advice and some training to be given on it”* (FG9, P1, Fire Service)

Several barriers to engaging in training were identified. These included lack of managerial support to attend training, lengthy journeys to attend training sessions, or inconveniently scheduled training. Despite these practical barriers, when asked about the prospect of online training, which would cut down on travel-related issues and could potentially be accessed at times convenient to participants, there was consensus that it was not an acceptable alternative to face-to-face approaches:

*“In-person is I think is the only way to go […] – just book a function room where there’s coffees and breaks and just people can naturally, like, get together and talk about a certain issue” (FG9, P3, AGS/RNLI/CFR)*

Crucially, it was suggested that such face-to-face learning would need to incorporate role play approaches. Role play was viewed across all focus groups as a powerful pedagogical tool, and much more effective than other approaches, such as PowerPoint presentations. For participants, role play sessions or scenario-based activities represented the “hands on” learning they yearned for in regard to suicide postvention:

*“You’ve got to have somebody in front of you playing the role of that relative that you’re trying to deal with you’re not going to do that looking at a PowerPoint or videos, it would have to be a face to face exercise”* (FG6, P4, NAS)

The significance of mandatory training was also expressed. Participants suggested that voluntary attendance at training is unlikely, as a result of competing commitments. Indeed, some participants referred to training programmes they were interested in completing but as these were not mandated, they felt they needed to prioritise other obligations. This indicates that, while first responders are aware of training programmes that would enhance their responses while attending suicide scenes, such programmes need to be mandated to ensure a greater uptake and integration into practice:

*“I think mandatory is a keyword there – I think if you don’t kind of push lads towards it, they won’t do it at all”* (FG6, P5, Fire Service)

Finally, participants spoke about training taking into account the perspectives of multiple first responders. Although some participants advocated for fully inclusive training, many others felt that clear role boundaries was a strength at the scene of a suicide, suggesting a more focused approach. Moreover, from a practical perspective, participants highlighted the challenges regarding delivering training to a single organisation, believing that co-ordinating multi-organisation training would be unfeasible. However, there was a profound respect demonstrated for the roles of other first responders at the scene of a suicide; as such, participants recommended that any training delivered should incorporate input from all relevant personnel:

*“I think maybe if there was a programme developed where it was, there should be an element of each agency’s roles within the programme”* (FG9, P1, Fire Service)

This final theme highlighted that, in the absence of formal training in suicide postvention communication, first responders often developed their skills through experience and by observing more experienced colleagues. Participants consistently identified a need for dedicated training, favouring face-to-face delivery over online approaches despite the practical advantages of the latter. They emphasised that training should be experiential and “hands on”, incorporating role play and practical scenarios to build confidence in communicating with bereaved family members. Participants also recommended that training programmes should reflect the roles and perspectives of all first responder organisations involved in attending suspected suicide scenes.

## 4. Discussion

This study explored the experiences and training needs of first responders when responding to the scene of a suspected suicide. The findings revealed that such scenes present challenging and emotionally charged environments for first responders, particularly surrounding postvention. Despite this, dedicated and prioritised training remains infrequent or non-existent, and the level and adequacy of support available in the aftermath is varied.

Survey findings indicated that first responders held positive attitudes towards suicide prevention and non-stigmatising attitudes towards suicidal persons. Similar findings were observed in the focus groups, where first responders spoke about the shift in attitudes and reduction of stigma which had occurred in recent years within their organisations, in the context of suicide. Positive attitudinal shifts for suicide have been associated with more positive response calls among law enforcement officers [39], showcasing the importances of improving first responders’ attitudes towards suicide. However, suicide literacy levels were moderate in terms of total LOSS scale scores suggest that while attitudes are encouraging, there remains room for improvement in first responders’ knowledge and understanding of the underlying mechanisms and theories within suicidology.

Preparedness is generally cited as a key element for an effective emergency response [40,41]. However, first responders in this study reported feeling inadequately prepared when attending the scene of a suspected suicide, especially when first joining a first responder organisation, with many focus group participants saying preparedness was not possible. While first responders, particularly members of AGS, relied heavily on guidelines and policies, and were generally confident in managing the procedural elements of the scene, communication with the bereaved was identified as a significant challenge. The struggles around communication with the bereaved on the scene is reflected in the low level of postvention knowledge revealed in the survey findings. This is particularly important as research shows that the first interaction of first responders with those bereaved can play a role in shaping their experience of loss and healing process [16]. Within the Irish context, first responders are often the first professionals to engage with families immediately following a suspected suicide [42]. Moreover, international evidence suggests that police officers feel unprepared to engage with families and deliver bad news, citing lack of training in this area as the reason for this [43]. Ensuring first responders are adequately equipped for postvention interactions can have a positive impact on outcomes for the bereaved, making it an area of critical importance for training and development. For example, training could introduce protocols used in healthcare, such as the SPIKES (Setting, Perception, Invitation, Knowledge, Emotions/Empathy, Strategy/Summary) protocol [44] to provide first responders with structured approaches to breaking bad news. The SPIKES protocol has been shown to increase confidence when combined with role play techniques. Employing such evidence-based approaches, in conjunction with role play, an experiential technique recommended by participants may support first responders in feeling more prepared when engaging with bereaved family members.

The aftermath of attending the scene also presented an array of challenges. The profound emotional toll of suicide and increased exposure was identified in this study, with 93% of first responders reporting that they had attended the scene of a suspected suicide. First responders spoke in detail about how the exposure had impacted them emotionally and how the high volume of calls can greatly impact first responders coping abilities over time, also cited as a prominent reason for resignation. This is consistent with research demonstrating that high exposure to suicide among first responders is associated with increased risk of adverse mental health outcomes, including depression, anxiety, post-traumatic stress disorder, and suicide [5,45,46]. Despite this, on average, first responders achieved low scores for organisational self-efficacy. Focus groups further highlighted the inconsistency of support available, with many viewing management supports as a “tick box” exercise. A high reliance on informal peer support was evident, with many also highlighting the benefit of post-incidence debriefs, which reflects previous work conducted in Ireland with first responders [47]. In the wake of exposure to traumatic events, support has been shown to aid processing, managing stressors and increasing wellbeing [48]. Therefore, organisations should place a greater emphasis on providing person-centred supports for first responder in the aftermath of responding to a suspected suicide.

Although over two thirds of participants reported having previously completed suicide-related training, the training and level of training which participants received was inconsistent and often deprioritised. Nonetheless, prior training was associated with improved literacy, attitudes, postvention knowledge, and preparedness, a finding which is consistent with the wider literature. However, the effect size for these findings were relatively low. This suggests that current training alone is unlikely to be sufficient and that there is a need for additional interventions. For example, multi-component interventions integrating education with meaningful engagement or lived experience narratives have been identified as effective strategies for improving attitudes and empathy and strengthening supportive responses [49]. In relation to postvention, breaking bad news training has been shown to increase confidence post-training, though this evidence is largely drawn from studies among medical students and hospital staff [50,51]. Other established suicide training programmes such as STORM (Skills Training on Risk Management) and ASIST (Applied Suicide Intervention Skills Training) have similarly been associated with improved attitudes towards suicide and increased confidence around suicide prevention [52,53].

While not statistically significant mandatory training yielded higher mean values for suicide literacy, attitudes, preparedness, and postvention knowledge, in comparison to non-mandatory training. This may suggest a greater impact when training is mandated which was reinforced in the focus groups, where participants emphasised the need for training to be mandated, consistent with evidence highlighting the absence of protected time as a key barrier to engagement with voluntary training [54]. Participants also raised important considerations around the format of training, specifically the need to weigh the merits of online versus in-person delivery to maximise the effectiveness of any training programme. These findings underscore the importance of incorporating first responders’ perspectives into the design and delivery of training programmes.

Lastly, in terms of training practicalities/modalities, there was support for face-to-face approaches over online ones. In a systematic review, Schmeckenbecher et al. examined the effect of e-learning suicide prevention training [55]. They found evidence to support the use of e-suicide prevention training, with medium effect size on knowledge, self-efficacy, and behaviour. However, they noted that effect of training on real world performance was not examined in any study, concluding that a combination of approaches using different training modalities including simulation-based training is likely to be beneficial, given the diversity and complexity of the skills required. An emerging interest in simulation-based training for mental health professionals [56] and mental health crisis including suicide prevention [57] is evident in the literature. For example, Richard et al. found preliminary evidence to suggest that simulation-based suicide prevention training may exert positive effects on behaviours and patient outcomes. Additionally, virtual and mixed reality simulations offer greater flexibility, allow for the inclusion of all types of first responders, and can be specifically designed to meet pre-defined learning outcomes. However, while there is obvious potential in these approaches the existing body of evidence indicates the need for more high-quality research [58].

## 5. Limitations

This study comprised a large nationally representative cross-sectional study of first responders in Ireland, complemented by insights from over 40 first responders through 10 multidisciplinary focus groups. However, this study is not without limitations. For both quantitative and qualitative components, the potential for self-selection bias must be acknowledged. Recruitment was conducted through first responder organisations, professional networks, and social media, meaning participation was voluntary. Consequently, participants may have had personal reasons for contributing, such as a particular interest in suicide prevention. Therefore, first responders who did not participate may have alternative perspectives on attending the scene of a suspected suicide. Additionally, because the survey was disseminated through multiple organisational mailing lists, professional networks, and social media platforms, the total number of individuals who received the survey invitation was unknown. Consequently, it was not possible to calculate an overall response rate.

In terms of the quantitative findings, the preparedness measure was developed specifically for this study. Although the scale demonstrated good internal consistency, it has not undergone formal psychometric validation. Consequently, findings relating to preparedness should be interpreted with caution. Results were not adjusted for profession to avoid reporting profession-specific findings and due to restrictions specified in our ethical approval; however, this may have introduced confounding regarding differences in terms of each professions’ suicide literacy, attitudes, postvention knowledge, and preparedness. Similarly, findings were not adjusted for duration and time since completion of training; this was due to the open text-nature exclusive to these questions, resulting in large amounts of missing data.

Lastly, as this study was conducted in Ireland, the findings should be interpreted in light of that country’s organisational structures, training provision, and policy context. Although many of the issues identified align with international literature, differences in first responder roles, service configuration, and postvention practices across countries may influence the transferability of these findings.

## 6. Implications of findings

The findings of this study hold significant implications for suicide related-training, organisation practice, and policy in relation to responding to the scene of a suspected suicide for first responders in Ireland. The associations between previous training and improved suicide literacy, attitudes, preparedness, and postvention knowledge underscore the importance of targeted training for first responders. The findings suggest that training should be mandatory, and multicomponent in line with evidence-based approaches.

The challenges identified around communication with bereaved family members highlight a clear need for postvention competencies to be embedded as a core element of first responders practice, alongside their procedural duties. Additionally, the variability in support to first responders suggest that organisations should consider moving beyond ad hoc support mechanisms, towards consistent, structure and person-centred supports, which encouraged debriefs.

At a policy level, there is a need for a nationally coordinated approach to first responders training for attending the scene of a suicide. The fragmentation and inconsistency of training provision and support structures across professions and organisations suggest inequitable arrangements. National guidance should be developed for suicide postvention training, communication with bereaved family members, and post-incident support to ensure that all first responders, regardless of profession or organisation, have access to adequate preparedness and support.

## 7. Conclusion

This nationally representative mixed-method study provides invaluable insights into the experiences and training needs of first responders responding to the scene of a suspected suicide. The findings revealed that first responders are regularly exposed to suicide and face considerably emotionally demanding scenes, particularly through the interaction with bereaved family members and communities. Despite this, there is inconsistent training and variable organisational support. Gaps in suicide literacy, preparedness and postvention knowledge highlight the need for training that is structured and mandatory. Equally, the wellbeing of first responders following attending the scene must be better supported through consistent and person-centred supports. Collectively, these findings call for coordinated training which places preparedness, communication with those bereaved, and wellbeing of first responders at its core, to improve outcomes for first responders and the bereaved family members they interact with.

## Supporting information

S1 File Participant breakdown by county. (DOCX)
