# Supplementary material for: First responders’ experiences and training needs at the scene of a suspected suicide in Ireland: A mixed methods study
Source: PLoS One. 2026 Oct 13;21(10):e0360382. doi: 10.1371/journal.pone.0360382 (PMC13660927; doi:10.1371/journal.pone.0360382)
Supplement: Supplementary file 1 [file pone.0360382.s001.docx]

| **County** | n (%) |
| --- | --- |
| Cork | 209 (13.3) |
| Carlow | 23 (1.5) |
| Cavan | 27 (1.7) |
| Clare | 44 (2.8) |
| Donegal | 50 (3.2) |
| Dublin | 436 (27.7) |
| Galway | 56 (3.6) |
| Kerry | 49 (3.1) |
| Kildare | 54 (3.4) |
| Kilkenny | 24 (1.5) |
| Laois | 32 (2.0) |
| Leitrim | 11 (0.7) |
| Longford | 16 (1.0) |
| Limerick | 56 (3.6) |
| Louth | 38 (2.4) |
| Mayo | 52 (3.3) |
| Meath | 34 (2.2) |
| Monaghan | 19 (1.2) |
| Offaly | 22 (1.4) |
| Roscommon | 14 (0.9) |
| Sligo | 27 (1.7) |
| Tipperary | 61 (3.9) |
| Waterford | 44 (2.8) |
| Westmeath | 24 (1.5) |
| Wexford | 51 (3.2) |
| Wicklow | 81 (5.1) |
| National/regional | 19 (1.2) |

**Supplementary File 1- Participant breakdown by county**
